# EUPopLink Country report - Serbia

**DOI:** 10.12688/openreseurope.23443.1

**Published:** 2026-05-07

**Authors:** Andrijana Lazarevic, Aleksandra Markovic

**Affiliations:** 1 Faculty of Political Sciences, University of Belgrade, Beograd, 11000, Serbia; 2 Faculty of Philosophy, University of Belgrade, Institute for Sociological Research, Beograd, 11000, Serbia

**Keywords:** populism, euroscepticism, Serbia

## Abstract

This country report, prepared as part of the EUPopLink COST Action, explores Serbia’s political dynamics in the context of European integration and Euroscepticism. It traces the rise of the Serbian Progressive Party (SNS) since 2012 and its impact on Serbia’s political landscape, with a focus on populist actors and their relationship with the EU. The report highlights the complex Euroscepticism in Serbia, where public support for EU membership has fluctuated and remains influenced by national sovereignty concerns, especially in relation to Kosovo. Populist parties, including SNS, Dveri, and Zavetnici, exhibit varying degrees of opposition to the EU, from pragmatic Euroscepticism to hard rejection, while balancing their domestic and foreign policy priorities. The analysis examines the shifting nature of populist Euroscepticism in Serbia, illustrating how it influences the country’s EU integration process, political discourse, and electoral strategies.

## 1. Introduction

The present country report was prepared as part of the EUPopLink COST Action ^1^ following the EUPopLink Theoretical Framework and guidelines. ^2^

European integration has been one of the most important and divisive issues in Serbian politics since the early 2000s. Accession negotiations officially began in January 2014, with 22 out of 35 chapters opened and two provisionally closed as of June 2025. While membership in the EU is formally presented as a strategic priority, progress has been repeatedly slowed by concerns over the rule of law, democracy, and media. ^3^ At the same time, the EU is Serbia’s main economic partner and donor, ^4, 5^ which makes the issue central not only in foreign policy but also in domestic debates. Public and political discussions are further shaped by Serbia’s balancing between the EU, Russia, USA and China, as well as by the unresolved question of Kosovo, which remains the most significant obstacle to accession.

Public opinion in Serbia exhibits a pronounced and complex form of Euroscepticism. Between 2009 and 2022, an average of 54.7% of respondents indicated support for EU membership in hypothetical referendum questions. ^6^ However, since 2022, no new polling or research on this topic has been conducted by the Serbian Ministry of European Integration. ^7^ According to the Balkan Barometer 2024 by the Regional Cooperation Council, only 34% of Serbian respondents believe that EU membership would be beneficial—the lowest level of support among Western Balkan countries—while 26% view it negatively, positioning *Serbia as the most EU-sceptical nation in the region.* By comparison, negative perceptions are far lower in Albania (2%), Kosovo (0%), and Montenegro (10%). Additionally, only 7% of Serbians expect accession by 2030 and 16% by 2035, while 35% believe Serbia will never join the EU. ^8^

This analysis concentrates on the post-2012 period, following the Serbian Progressive Party’s (SNS) rise to power, a development that significantly reshaped Serbia’s political dynamics. Since 2012, SNS has dominated Serbian politics, shaping government policies and the broader political discourse. Additionally, analysis will be limited to the relevant parliamentary parties for this paper, given that Serbia has more than 130 registered political parties, ^9^ a highly fragmented party system where most parties remain small and marginal.

## 2. Populist political actors

In mainstream Serbian politics, there are currently ***no parliamentary parties that fully embody left-wing populism*** as defined by a rejection of contemporary capitalism and advocacy for major redistribution from political elites.

### Valence populist parties

• **The ruling Serbian Progressive Party (SNS)**, effectively led by Aleksandar Vučić, the President of the Republic of Serbia, can be described as a *patrimonial party* due to Vučić’s significant centralization of power and control over both party structures and state resources. This patrimonial characteristic is evident in the party’s extensive influence over state institutions and resources, which it leverages to maintain loyalty and political dominance, thereby blurring the lines between the party and the state. SNS merges ***valence populism*** with elements of nationalism, authoritarianism, and a soft Eurosceptic stance. ^10^ The party employs populist strategies, including the personalization of power around Vučić as the nation’s protector, anti-elitist rhetoric, and economic populism through pre-election social initiatives. ^11, 12^
  SNS’s *populism is flexible, pragmatic and catch-all* rather than ideologically rigid, emphasizing moral integrity and anti-corruption while maintaining a pro-EU stance in principle but with Eurosceptic elements. SNS is an associate member of the European People’s Party (EPP), reflecting its moderate and pragmatic European alignment. SNS has not joined transnational far-right or radical populist networks but rather positions itself as a stabilizing force with a nationalist-populist profile adapted to Serbia’s context.
• **Dosta je Bilo (DJB)**, led by Saša Radulović, exhibits a distinctly populist approach characterized by its anti-elite and anti-corruption stance, often framed as an “anti-party” formation. Although no longer a relevant political force, DJB played a formative role in introducing antipolitics as a mode of political contestation that became central in Serbia after 2012. The party’s populism emphasizes economic self-reliance, a strong opposition to political elites, and a rejection of perceived European Union interference in Serbian politics. DJB effectively channels popular dissatisfaction with the political establishment and globalization, prioritizing principles of transparency and accountability. Unlike parties that focus heavily on ideological or identity-based issues, DJB’s populism centers more on *valence issues* - such as combating corruption and advocating for democratic reform. This positions DJB within the broader spectrum of valence populism, where the focus is on moral integrity and anti-establishment rhetoric rather than on radical socio-economic or nationalist agendas.
• **The Socialist Party of Serbia (SPS)** is a populist party with a historically nationalist-authoritarian legacy, currently characterized by a *valence populist style.* Under the leadership of Slobodan Milošević, during 1990s, SPS merged nationalist rhetoric with authoritarian practices and populist nationalism. This era was marked by a monopolistic party structure, a strong concentration of power, and an anti-Western, anti-liberal stance, However, the nationalism espoused by SPS has been more pragmatic and opportunistic than strictly ideological nativism, distinguishing it from classic populist radical right parties, particularly when compared to the Serbian Radical Party (SRS, the most extreme right-wing party, and ruling SNS emerged from this party (for more ^13^). Since Ivica Dačić took over leadership, SPS has transitioned towards a more moderate stance, while still retaining a populist rhetoric. Today, its populism is more valence-oriented, advocating for nationalist-conservative and socialist ideologies, yet it does not fundamentally reject capitalism or call for radical redistribution. Regime-affiliated parties, such as the SPS, tend to appeal to an older demographic that is inclined toward authoritarianism, often characterized by a reliance on government-controlled media. These citizens typically hold socially conservative perspectives. Economically, they support a degree of redistribution and state regulation; however, their beliefs do not exhibit a strong ideological rigidity. ^14^

### Populist radical right-wing parties in Serbia

• **Dveri (Serbian Movement Dveri)**, led by Boško Obradović, is a radical right populist party that adopts a hardline stance against the EU and globalization. Dveri articulates its populism by framing society as divided between the “pure people” (ethnic Serbs, traditional families, and Christian values) and a corrupt elite, which includes the EU, migrants, LGBTQ+ activists, and liberal politicians. Dveri’s populism is further reflected in its opposition to globalization and the EU, which they depict as external forces that undermine Serbian sovereignty and traditional values. Their emphasis on “family values”, anti-immigration stances, and economic nationalism serves to bolster their populist appeal by addressing perceived threats to the native group and promising to restore social order and national pride. Dveri is affiliated with far-right European networks, most notably drawing inspiration from Germany’s Alternative für Deutschland (AfD). ^15^
• **Zavetnici (Oathkeepers)**, under the leadership of Milica Đurđević Stamenkovski, embody far-right populism with a strong pro-Russian and anti-EU perspective. They reject NATO and the EU, employing the populist rhetoric to assert that they represent the genuine Serbian people against a corrupt elite, whom they accuse of “selling out” national sovereignty, particularly concerning Kosovo. Their pro-Russian and anti-EU positions are framed as a defense of Serbia’s identity and independence from foreign influences, appealing to populist themes of national sovereignty and anti-globalism. Their focus on identity politics and sovereignty aligns them with European far-right parties such as Germany’s Alternative for Germany (AfD) and Hungary’s Our Homeland Movement, from which they have garnered electoral support. ^16, 17^

In 2023, Zavetnici entered into a coalition with the right-wing movement Dveri under the joint list **“Nacionalno okupljanje” (National Gathering)**. Sharing nationalism, traditional values, and opposition to EU integration, the coalition advanced a sharply anti-Western platform, rejecting Kosovo’s independence, Serbia’s accession to the EU and NATO, and promoting closer ties with Russia, China, and BRICS. Their transnational far-right connections included support from Alternative für Deutschland (AfD), as well as cooperation with Hungary’s *Mi Hazánk Mozgalom* and Bulgaria’s *Vazrazhdane* (Revival). Although the coalition failed to surpass the electoral threshold in December 2023, Zavetnici soon shifted towards pragmatic realignment, joining the SNS-led government in 2024 and moderating its rhetoric into a discourse of “strategic neutrality”.

• **Branimir Nestorović** has emerged as one of the most striking figures of Serbia’s contemporary populist scene, blending anti-elite rhetoric, conspiracy theories, and nationalist symbolism into a distinctive political brand. A former pulmonologist and controversial public figure during the COVID-19 pandemic—where he gained attention for dismissing the virus and promoting unscientific claims—Nestorović now channels widespread distrust in institutions into a populist narrative centered on “common sense” national sovereignty, and the will of “the people.” His movement, Mi – Snaga naroda (We – Power of the People), positions itself as an authentic voice against corrupt elites, globalist influences, and perceived Western ideological impositions. Nestorović’s populism is characterized by a deep skepticism of expert authority, emotional appeals to tradition and identity, and a Manichean worldview in which ordinary citizens must “take back” the country from both domestic and foreign conspirators. Through this discourse, he attracts voters disillusioned with both the ruling SNS and liberal opposition, offering a simplified, anti-system alternative rooted in cultural conservatism and anti-globalist sentiment. The movement gained 5 seats in the National Assembly after the December 2023 parliamentary elections and 10 seats in the 2024 Belgrade City Assembly, establishing itself as a small but notable populist force. ^18, 19^

## 3. Positions on ‘europe’ and the populism-euroscepticism nexus

Several Serbian PRR parties, including Dveri, Zavetnici, and the Serbian Radical Party (SRS), express strong opposition to both the principle and practice of the European Union, demonstrating ***hard Euroscepticism***. They reject the EU’s institutional framework and specific policies, particularly those perceived as *threats to Serbian sovereignty*, national identity, and traditional values. This perspective is particularly pronounced when it comes to EU pressure regarding Kosovo’s status, migration, and LGBTQ+ rights. These parties characterize the EU as a corrupt elite that imposes policies detrimental to the national interests of Serbia. Additionally, they critique the EU’s future trajectory, viewing it as a supranational entity that undermines national identities and traditional values. These parties usually advocate for enhanced diplomatic and economic relations with Russia while simultaneously rejecting the North Atlantic Treaty Organization (NATO).

Dveri advocate closer alignment with Russia and their stance has remained consistent, with little evidence of moderation in response to EU-related developments. Zavetnici also adopt hard Euroscepticism. Prior to 2022, their rhetoric positioned them as uncompromising defenders of sovereignty. However, following their integration into the SNS-led government in 2024, explicit rejection of EU membership was replaced with discourse around “national interest” and “strategic neutrality”. This illustrates a shift from hard Euroscepticism toward a more opportunistic, moderated position once the party entered mainstream structures. The Serbian Radical Party represents the longest-standing form of hard Euroscepticism in Serbia. Rooted in an ultranationalist tradition, the party rejects both the EU and NATO as fundamentally hostile to Serbian sovereignty, consistently advocating for closer ties with Russia. Its discourse has been shaped by opposition to the ICTY (International Criminal Tribunal for the former Yugoslavia), Kosovo’s independence, and EU liberal norms. Unlike other actors, SRS has not moderated its position over time, but its declining electoral relevance limits its current impact on the Eurosceptic debate.

Dosta je Bilo initially articulated soft Euroscepticism framed through anti-elite and anti-corruption rhetoric rather than outright rejection of the EU. While not rejecting EU integration in principle, DJB depicted the EU as another elite structure disconnected from citizens’ needs. Although it has since faded from political relevance, DJB’s legacy lies in normalizing antipolitical frames of Euroscepticism that blurred the line between pro- and anti-EU discourse.

The ruling SNS’s Euroscepticism is informed by its broader ideological profile as a catch-all, valence populist party mixing nationalism, authoritarianism, and economic pragmatism. Its criticism of the EU often aligns with defending national sovereignty and resisting external pressures perceived as threats to Serbia’s political autonomy, while simultaneously leveraging EU accession as a legitimizing framework. This ***pragmatic soft Euroscepticism*** allows SNS to maintain associate membership in the European People’s Party (EPP) and present itself as pro-European internationally, even as it uses soft Eurosceptic rhetoric domestically to appeal to nationalist and skeptical segments of the electorate.

Concerning the object of opposition, SNS does not fundamentally reject the concept or principle of European integration. Rather, it articulates *conditional criticism*, primarily aimed at the EU’s institutional practices and policies, particularly those viewed as encroaching on Serbia’s sovereignty or requiring politically sensitive concessions, such as the normalization of relations with Kosovo. SNS’s vision of the future is pragmatic: Serbia as a bridge between East and West, cooperating with the EU while preserving strategic partnerships with Russia and China, maintaining military neutrality, and avoiding full alignment with Western security structures like NATO.

The populism of the Socialist Party of Serbia (SPS) is distinguished by its flexible and pragmatic orientation, which integrates pro-European integration rhetoric with a measured, occasionally Eurosceptic perspective. This is most evident in its emphasis on sovereignty and resistance to Kosovo-related conditionality, while simultaneously participating in coalition governments that formally support EU integration. SPS’s flexibility allows it to adapt its discourse depending on coalition arrangements, positioning itself as both pro-European and nationalist.

## 4. Responses to euroscepticism and consequences

Mainstream pro-European Union parties in Serbia, particularly those in government, have largely addressed Eurosceptic discourses in a conciliatory and ambiguous manner. They selectively embrace or downplay Eurosceptic rhetoric to expand their appeal, prevent voter polarization, and uphold coalition stability. Adversarial responses are infrequent, and outright rejection is uncommon, given the political significance of the issue. ^20^

Genuinely pro-EU parties in Serbia, such as the Party of Freedom and Justice (SSP), the Green–Left Front (ZLF), and remnants of the Democratic Party (DS), respond to rising Euroscepticism primarily through normative reaffirmation of European values, linking EU integration to democratic consolidation, judicial reform, and media freedom. Rather than engaging in emotional or populist counter-narratives, these actors emphasize institutional and rule-of-law frameworks as the foundation for credible accession. Their criticism is often twofold: directed at the Serbian government for undermining reforms, and at the EU for tolerating democratic backsliding in exchange for regional stability. As such, they adopt what could be described as ***adversarial strategies***, positioning themselves in critical opposition to anti-EU narratives while supporting integration - firmly supportive of integration, yet vocal about the gap between EU rhetoric and its engagement with the current regime. However, the consequences of these responses remain limited due to the fragmentation of the pro-European camp, reduced media access, and weak electoral performance. Unlike mainstream actors in other EU-aspiring states, Serbian pro-European parties cannot effectively shape the national narrative on Europe. Their discourse often resonates only within pro-democracy civil society circles and urban constituencies. One notable example of contested EU-related policy concerns lithium exploitation and associated environmental and social risks, which has become a highly salient narrative in the Eurosceptic debate and a point of critique by pro-European actors attempting to reconcile development with democratic and ecological principles.

Mainstream reactions to populist Euroscepticism are constrained by the dominance of the Serbian Progressive Party (SNS), which strategically oscillates between pro-European and sovereigntist discourses. Thus, the response pattern is neither clearly adversarial nor fully accommodative. Instead, SNS neutralizes Eurosceptic actors by co-opting elements of their rhetoric and occupying the full ideological spectrum. It reflects a ***variation of the accommodative strategy***, as the party strategically oscillates between pro-European and sovereigntist discourses to maintain political dominance and broaden its electoral appeal. The concrete consequences of Euroscepticism in Serbia are mostly rhetorical. SNS frequently uses EU criticism to deflect accountability, particularly on rule-of-law issues and Kosovo negotiations. Nonetheless, it continues to seek EU funds, participate in accession negotiations, and maintain a technocratic administrative structure to ensure alignment with EU standards in key policy areas.

Hard Eurosceptic actors, such as Dveri and, increasingly, the New Democratic Party of Serbia (Novi DSS), have minimal influence on policymaking. Their presence functions more as a symbolic signal to Brussels that public dissatisfaction exists, but they remain excluded from governing coalitions. Overall, Euroscepticism is a secondary political theme ( ***dismissive strategy***), typically mobilised during election campaigns or crises, but ultimately subordinated to broader nationalist narratives and regime survival strategies.

Zavetnici initially exemplified an adversarial Eurosceptic stance, vocally opposing the EU and NATO while advocating pro-Russian positions before 2022. However, after joining the SNS-led government in 2024, they moderated their rhetoric toward appeals to “national interest” and “strategic neutrality”, reflecting an accommodative shift aligned with mainstream coalition narratives. This change demonstrates how *access to governing structures can discipline and instrumentalise populist Euroscepticism in Serbia.*

## 5. Short conclusion

Populist Euroscepticism in Serbia is state-centric, sovereigntist, and highly instrumental. The Serbian case demonstrates the fluid and performative nature of populist Euroscepticism in non-EU contexts. Unlike EU member states, where Euroscepticism implies contestation from within, in Serbia it often combines Eurosceptic messaging with a pragmatic engagement toward the EU. This engagement does not reflect a genuine commitment to European values, but rather a strategic pursuit of economic and political benefits associated with EU accession. While multiple issues inform Eurosceptic rhetoric, the question of Kosovo represents a central and unifying reference point for all major populist actors, shaping the contours of critique toward both domestic and European actors (Kovačević and Surlić, 2025). This hybrid model of simultaneous integration and resistance complicates efforts to categorize Serbian populist actors neatly and calls for a more nuanced understanding of Euroscepticism in accession countries.

## Ethics and consent

Ethical approval and consent were not required.

## Data Availability

No new data were generated or analysed in this manuscript. All data are derived from publicly available sources, which are cited in the reference list.

## References

[1] Andreadis I Vasilopoulou S : The populism-euroscepticism nexus in a contested Europe: The EUPopLink COST Action [version 1; peer review: 3 approved]. *Open Res Europe.* 2026;6:27. 10.12688/openreseurope.22680.1 PMC12910195 41710161

[2] Lorimer M Roch J Kessel S van : Populism, euroscepticism, and the populism–euroscepticism nexus [version 1; peer review: awaiting peer review]. *Open Res Europe.* 2026;6:73. 10.12688/openreseurope.23109.1 PMC13560596 42728954

[3] European Commission: Serbia 2024 Report (SWD(2024) 695 final). 2024. Reference Source

[4] Delegation of the European Union to the Republic of Serbia: EU assistance to Serbia. Reference Source

[5] Delegation of the European Union to the Republic of Serbia: Trade relations between the EU and Serbia. Reference Source

[6] Ministry of European Integration: European orientation of citizens of Serbia: Public opinion poll (December 2022). 2023. Reference Source

[7] Ministry of European Integration: Public opinion research.(accessed March 2026). Reference Source

[8] Regional Cooperation Council: Balkan Barometer 2024: Key findings. 2024. Reference Source

[9] Ministry of Public Administration and Local Self-Government: Register of political parties.(accessed March 2026). Reference Source

[10] Spasojević D : Balancing on a pin: Serbian populists, the European Union and Russia. *The impacts of the Russian invasion of Ukraine on right-wing populism in Europe.* European Center for Populism Studies;2023. p.266–277. Reference Source

[11] Bieber F : Patterns of competitive authoritarianism in the Western Balkans. *East European Politics.* 2018;34(3):337–354. 10.1080/21599165.2018.1490272

[12] Kovačević D Surlić S : Populist patterns in post-conflict settlement: leadership discursive strategies in the Serbia-Kosovo status dispute. *Southeast European and Black Sea Studies.* 2025;1–19. 10.1080/14683857.2025.2565869

[13] Konitzer A : Speaking European: Conditionality, public attitudes and pro-European party rhetoric in the Western Balkans. *Eur. Asia Stud.* 2011;63(10):1853–1888. 10.1080/09668136.2011.618699

[14] Jakšić I Gajić Đ Vlajnić N : How partisan media exposure shaped party evaluations in the 2023 elections in Serbia. *Politički život: časopis za analizu politike.* 2023;26:49–79. 10.18485/fpn_pz.2024.26.4

[15] Dveri: Search results for “Alternative za Nemačku”.(accessed March 2026). Reference Source

[16] FoNet: Podrška za AfD.(accessed March 2026). Reference Source

[17] Plus Online: Predstavnici Srpske stranke Zavetnici prisustvovali međunarodnoj konferenciji u Sofiji “Novi lideri Evrope”i posetili KSB bugarsku građevinsku komoru. 2024. (accessed March 2026). Reference Source

[18] National Assembly of the Republic of Serbia: National Assembly in numbers.(accessed March 2026). Reference Source

[19] City of Belgrade: City Election Commission adopted the decision on preliminary results of the elections for councillors of the Belgrade City Assembly. 2024. (accessed March 2026). Reference Source

[20] Styczyńska N Dajč H : Between the past and the future: Eurosceptic political parties and Serbia. Sondel-Cedarmas J Berti F , editors. *The right-wing critique of Europe: Nationalist, sovereignist and right-wing populist attitudes to the EU.* Routledge;2022. 10.4324/9781003226123

