# EUPopLink Country report - Serbia

**DOI:** 10.12688/openreseurope.23443.2

**Published:** 2026-09-16

**Authors:** Andrijana Lazarevic, Aleksandra Markovic

**Affiliations:** 1 Faculty of Political Sciences, University of Belgrade, Beograd, 11000, Serbia; 2 Faculty of Philosophy, University of Belgrade, Institute for Sociological Research, Beograd, 11000, Serbia

**Keywords:** populism, euroscepticism, Serbia

## Abstract

This country report, prepared as part of the EUPopLink COST Action, explores Serbia’s political dynamics in the context of European integration and Euroscepticism. It traces the rise of the Serbian Progressive Party (SNS) since 2012 and its impact on Serbia’s political landscape, with a focus on populist actors and their relationship with the EU. The report highlights the complex Euroscepticism in Serbia, where public support for EU membership has fluctuated and remains influenced by national sovereignty concerns, especially in relation to Kosovo. Populist parties, including SNS, Dveri, and Zavetnici, exhibit varying degrees of opposition to the EU, from pragmatic Euroscepticism to hard rejection, while balancing their domestic and foreign policy priorities. The analysis examines the shifting nature of populist Euroscepticism in Serbia, illustrating how it influences the country’s EU integration process, political discourse, and electoral strategies.

## 1. Introduction

The present country report was prepared as part of the EUPopLink COST Action ^1^ following the EUPopLink Theoretical Framework and guidelines. ^2^

European integration has been one of the most important and divisive issues in Serbian politics since the early 2000s. Accession negotiations officially began in January 2014, with 22 out of 35 chapters opened and two provisionally closed as of June 2025. While membership in the EU is formally presented as a strategic priority, progress has been repeatedly slowed by concerns over the rule of law, democracy, and media. ^3^ At the same time, the EU is Serbia’s main economic partner and donor, ^4, 5^ which makes the issue central not only in foreign policy but also in domestic debates. Public and political discussions are further shaped by Serbia’s balancing between the EU, Russia, USA and China, as well as by the unresolved question of Kosovo, which remains the most significant obstacle to accession.

Public opinion in Serbia exhibits a pronounced and complex form of Euroscepticism. Between 2009 and 2022, an average of 54.7% of respondents indicated support for EU membership in hypothetical referendum questions. ^6^ However, since 2022, no new polling or research on this topic has been conducted by the Serbian Ministry of European Integration. ^7^ According to the Balkan Barometer 2024 by the Regional Cooperation Council, only 34% of Serbian respondents believe that EU membership would be beneficial—the lowest level of support among Western Balkan countries, while 26% view it negatively, positioning *Serbia as the most EU-sceptical nation in the region.* By comparison, negative perceptions are far lower in Albania (2%), Kosovo (0%), and Montenegro (10%). Additionally, only 7% of Serbians expect accession by 2030 and 16% by 2035, while 35% believe Serbia will never join the EU. ^8^ These figures should not be interpreted as evidence of a single or uniform public attitude. Rather, they indicate the coexistence of support for the economic and political benefits associated with the EU and opposition to specific forms of conditionality, particularly those connected with Kosovo, sovereignty and foreign-policy alignment.

This analysis concentrates on the post-2012 period, following the Serbian Progressive Party’s (SNS) rise to power, a development that significantly reshaped Serbia’s political dynamics. The choice of 2012 as the starting point does not imply a sudden reversal of Serbia’s foreign-policy orientation or the immediate emergence of Euroscepticism. Rather, 2012 is treated as an analytical turning point because the Serbian Progressive Party (SNS) entered government and subsequently became the dominant political actor. This transformation affected the structure of political competition, the relationship between party and state institutions, and the way populist and Eurosceptic claims were incorporated into mainstream government discourse. The period is therefore examined as one of institutionalised and strategically managed populism, in which formal support for EU integration coexists with sovereigntist criticism of the EU.

The selection of actors follows three criteria: parliamentary representation during the period under discussion, electoral relevance and analytical relevance to the populism–Euroscepticism nexus. The report therefore includes dominant governing actors, parliamentary opposition parties, historically influential parties and smaller actors whose importance is primarily discursive. Because parties' parliamentary status and electoral relevance have changed over time, the report distinguishes between current political weight and broader relevance to the development of populist Euroscepticism. This approach is necessary in a highly fragmented party system in which Serbia has more than 130 registered political parties (Ministry of Public Administration and Local Self-Government, n.d.). In contrast, most parties remain small or marginal.

The electoral context helps to calibrate the political significance of the actors discussed below. In the December 2023 parliamentary elections, the SNS-led list won 129 of the 250 seats with 46.75% of the vote. The opposition list Serbia Against Violence won 65 seats, the Socialist Party of Serbia (SPS) won 18, the NADA coalition won 13, and Branimir Nestorović’s list We - the Voice of the People won 13 seats. Five minority lists also entered the National Assembly (National Assembly – Republic of Serbia, n.d.). Dveri and Zavetnici, which had previously represented important radical-right and Eurosceptic positions, did not enter parliament as a joint list in those elections. This distribution demonstrates the difference between electoral dominance, parliamentary representation and discursive influence. Explicitly hard Eurosceptic actors may remain electorally limited, while the dominant governing party can incorporate selected sovereigntist frames into a much broader electoral coalition.

## 2. Populist political actors

In mainstream Serbian politics, there are currently *no parliamentary parties that fully embody left-wing populism* as defined by a rejection of contemporary capitalism and advocacy for major redistribution from political elites. This does not mean that left-wing or culturally progressive political actors are absent. The Green–Left Front (ZLF), for example, combines progressive positions on social and environmental issues with criticism of corruption, clientelism and elite domination. However, its political profile and programme do not consistently construct a populist opposition between a homogeneous people and a uniformly defined elite in the same way as the radical-right actors discussed below.

### Valence populist parties

• **The ruling Serbian Progressive Party (SNS)**, effectively led by Aleksandar Vučić, the President of the Republic of Serbia, can be described as a *patrimonial party* due to Vučić’s significant centralization of power and control over both party structures and state resources. This patrimonial characteristic is evident in the party’s extensive influence over state institutions and resources, which it leverages to maintain loyalty and political dominance, thereby blurring the lines between the party and the state. SNS merges *valence populism* with elements of nationalism, authoritarianism, and a soft Eurosceptic stance. ^10^ The party employs populist strategies, including the personalization of power around Vučić as the nation’s protector, anti-elitist rhetoric, and economic populism through pre-election social initiatives. ^11, 12^
  SNS’s *populism is flexible, pragmatic and catch-all* rather than ideologically rigid, emphasizing moral integrity and anti-corruption while maintaining a pro-EU stance in principle but with Eurosceptic elements. SNS is an associate member of the European People’s Party (EPP), reflecting its moderate and pragmatic European alignment. SNS has not joined transnational far-right or radical populist networks but rather positions itself as a stabilizing force with a nationalist-populist profile adapted to Serbia’s context. Its electoral dominance gives this hybrid profile greater practical significance than the positions of smaller parties that articulate more explicit opposition to the EU.
• **Dosta je Bilo (DJB)**, led by Saša Radulović, exhibits a distinctly populist approach characterized by its anti-elite and anti-corruption stance, often framed as an “anti-party” formation. Although no longer a relevant political force, DJB played a formative role in introducing antipolitics as a mode of political contestation that became central in Serbia after 2012. The party’s populism emphasizes economic self-reliance, a strong opposition to political elites, and a rejection of perceived European Union interference in Serbian politics. DJB effectively channels popular dissatisfaction with the political establishment and globalization, prioritizing principles of transparency and accountability. Unlike parties that focus heavily on ideological or identity-based issues, DJB’s populism centers more on *valence issues* - such as combating corruption and advocating for democratic reform. This positions DJB within the broader spectrum of valence populism, where the focus is on moral integrity and anti-establishment rhetoric rather than on radical socio-economic or nationalist agendas.
• **The Socialist Party of Serbia (SPS)** is a populist party with a historically nationalist-authoritarian legacy, currently characterized by a *valence populist style.* Under the leadership of Slobodan Milošević, during 1990s, SPS merged nationalist rhetoric with authoritarian practices and populist nationalism. This era was marked by a monopolistic party structure, a strong concentration of power, and an anti-Western, anti-liberal stance, However, the nationalism espoused by SPS has been more pragmatic and opportunistic than strictly ideological nativism, distinguishing it from classic populist radical right parties, particularly when compared to the Serbian Radical Party (SRS, the most extreme right-wing party, and ruling SNS emerged from this party (for more ^13^). Since Ivica Dačić took over leadership, SPS has transitioned towards a more moderate stance, while still retaining a populist rhetoric. Today, its populism is more valence-oriented, advocating for nationalist-conservative and socialist ideologies, yet it does not fundamentally reject capitalism or call for radical redistribution. Regime-affiliated parties, such as the SPS, tend to appeal to an older demographic that is inclined toward authoritarianism, often characterized by a reliance on government-controlled media. These citizens typically hold socially conservative perspectives. Economically, they support a degree of redistribution and state regulation; however, their beliefs do not exhibit a strong ideological rigidity. ^14^

### Populist radical right-wing parties in Serbia

• **Dveri (Serbian Movement Dveri)**, led by Boško Obradović, is a radical right populist party that adopts a hardline stance against the EU and globalization. Dveri articulates its populism by framing society as divided between the “pure people” (ethnic Serbs, traditional families, and Christian values) and a corrupt elite, which includes the EU, migrants, LGBTQ+ activists, and liberal politicians. Dveri’s populism is further reflected in its opposition to globalization and the EU, which they depict as external forces that undermine Serbian sovereignty and traditional values. Their emphasis on “family values”, anti-immigration stances, and economic nationalism serves to bolster their populist appeal by addressing perceived threats to the native group and promising to restore social order and national pride. Dveri is affiliated with far-right European networks, most notably drawing inspiration from Germany’s Alternative für Deutschland (AfD). ^15^
• **Zavetnici (Oathkeepers)**, under the leadership of Milica Đurđević Stamenkovski, embody far-right populism with a strong pro-Russian and anti-EU perspective. They reject NATO and the EU, employing the populist rhetoric to assert that they represent the genuine Serbian people against a corrupt elite, whom they accuse of “selling out” national sovereignty, particularly concerning Kosovo. Their pro-Russian and anti-EU positions are framed as a defense of Serbia’s identity and independence from foreign influences, appealing to populist themes of national sovereignty and anti-globalism. Their focus on identity politics and sovereignty aligns them with European far-right parties such as Germany’s Alternative for Germany (AfD) and Hungary’s Our Homeland Movement Mi Hazánk Mozgalom and Bulgaria’s Vazrazhdane (Revival) from which they have garnered electoral support. ^16, 17^

In 2023, Zavetnici entered into a coalition with the right-wing movement Dveri under the joint list **“Nacionalno okupljanje” (National Gathering)**. Sharing nationalism, traditional values, and opposition to EU integration, the coalition advanced a sharply anti-Western platform, rejecting Kosovo’s independence, Serbia’s accession to the EU and NATO, and promoting closer ties with Russia, China, and BRICS. Their transnational far-right connections included support from Alternative für Deutschland (AfD), as well as cooperation with Hungary’s *Mi Hazánk Mozgalom* and Bulgaria’s *Vazrazhdane* (Revival). Although the coalition failed to surpass the electoral threshold in December 2023, Zavetnici soon shifted towards pragmatic realignment, joining the SNS-led government in 2024 and moderating its rhetoric into a discourse of “strategic neutrality”. This trajectory illustrates the importance of institutional position: opposition status enabled a harder Eurosceptic profile, whereas access to government encouraged discursive moderation.

• **Branimir Nestorović** has emerged as one of the most striking figures of Serbia’s contemporary populist scene, blending anti-elite rhetoric, conspiracy theories, and nationalist symbolism into a distinctive political brand. A former pulmonologist and controversial public figure during the COVID-19 pandemic, where he gained attention for dismissing the virus and promoting unscientific claims, Nestorović now channels widespread distrust in institutions into a populist narrative centered on “common sense” national sovereignty, and the will of “the people.” His movement, Mi - Snaga naroda (We - Power of the People), positions itself as an authentic voice against corrupt elites, globalist influences, and perceived Western ideological impositions. Nestorović’s populism is characterized by a deep skepticism of expert authority, emotional appeals to tradition and identity, and a Manichean worldview in which ordinary citizens must “take back” the country from both domestic and foreign conspirators. Through this discourse, he attracts voters disillusioned with both the ruling SNS and liberal opposition, offering a simplified, anti-system alternative rooted in cultural conservatism and anti-globalist sentiment. The movement gained 13 seats in the National Assembly after the December 2023 parliamentary elections and 10 seats in the 2024 Belgrade City Assembly, establishing itself as a small but notable populist force. ^18, 19^ Its parliamentary representation distinguishes it from purely extra-parliamentary actors, although its overall influence remains considerably smaller than that of SNS.
• The New Democratic Party of Serbia (Novi DSS) is a parliamentary conservative-nationalist opposition party whose Euroscepticism is more explicit than that of SNS but less electorally dominant than that of the governing party. It is included because it represents an important bridge between established parliamentary opposition and harder sovereigntist positions on EU integration, Kosovo and foreign-policy alignment. Its presence also demonstrates that Eurosceptic and sovereigntist claims are not restricted to newly formed radical-right movements but can also be articulated by established parliamentary actors.

## 3. Positions on ‘europe’ and the populism-euroscepticism nexus

The Serbian radical-right parties discussed in this report articulate hard Euroscepticism by linking opposition to the EU with the defence of national sovereignty, traditional values and territorial claims concerning Kosovo. Their positions differ, however, in their degree of organisational persistence and political relevance. Dveri have maintained a relatively consistent anti-EU and pro-Russian orientation, while Zavetnici moderated their position after entering government. The Serbian Radical Party represents the most enduring form of hard Euroscepticism, although its declining electoral relevance limits its current political impact.

Dveri advocate closer alignment with Russia and their stance has remained consistent, with little evidence of moderation in response to EU-related developments. Zavetnici also adopt hard Euroscepticism. Prior to 2022, their rhetoric positioned them as uncompromising defenders of sovereignty. However, following their integration into the SNS-led government in 2024, explicit rejection of EU membership was replaced with discourse around “national interest” and “strategic neutrality”. This illustrates a shift from hard Euroscepticism toward a more opportunistic, moderated position once the party entered mainstream structures. The Serbian Radical Party represents the longest-standing form of hard Euroscepticism in Serbia. Rooted in an ultranationalist tradition, the party rejects both the EU and NATO as fundamentally hostile to Serbian sovereignty, consistently advocating for closer ties with Russia. Its discourse has been shaped by opposition to the ICTY (International Criminal Tribunal for the former Yugoslavia), Kosovo’s independence, and EU liberal norms. Unlike other actors, SRS has not moderated its position over time, but its declining electoral relevance limits its current impact on the Eurosceptic debate.

Dosta je Bilo initially articulated soft Euroscepticism framed through anti-elite and anti-corruption rhetoric rather than outright rejection of the EU. While not rejecting EU integration in principle, DJB depicted the EU as another elite structure disconnected from citizens’ needs. Although it has since faded from political relevance, DJB’s legacy lies in normalizing antipolitical frames of Euroscepticism that blurred the line between pro- and anti-EU discourse.

The ruling SNS’s Euroscepticism is informed by its broader ideological profile as a catch-all, valence populist party mixing nationalism, authoritarianism, and economic pragmatism. Its criticism of the EU often aligns with defending national sovereignty and resisting external pressures perceived as threats to Serbia’s political autonomy, while simultaneously leveraging EU accession as a legitimizing framework. This *pragmatic soft Euroscepticism* allows SNS to maintain associate membership in the European People’s Party (EPP) and present itself as pro-European internationally, even as it uses soft Eurosceptic rhetoric domestically to appeal to nationalist and skeptical segments of the electorate.

SNS does not fundamentally reject the concept or principle of European integration. Rather, it articulates *conditional criticism*, primarily aimed at the EU’s institutional practices and policies, particularly those viewed as encroaching on Serbia’s sovereignty or requiring politically sensitive concessions, such as the normalization of relations with Kosovo. SNS’s vision of the future is pragmatic: Serbia as a bridge between East and West, cooperating with the EU while preserving strategic partnerships with Russia and China, maintaining military neutrality, and avoiding full alignment with Western security structures like NATO.

The populism of the Socialist Party of Serbia (SPS) is distinguished by its flexible and pragmatic orientation, which integrates pro-European integration rhetoric with a measured, occasionally Eurosceptic perspective. This is most evident in its emphasis on sovereignty and resistance to Kosovo-related conditionality, while simultaneously participating in coalition governments that formally support EU integration. SPS’s flexibility allows it to adapt its discourse depending on coalition arrangements, positioning itself as both pro-European and nationalist.

## 4. Responses to euroscepticism and consequences

Mainstream pro-European Union parties in Serbia, particularly those in government, have largely addressed Eurosceptic discourses in a conciliatory and ambiguous manner. They selectively embrace or downplay Eurosceptic rhetoric to expand their appeal, prevent voter polarization, and uphold coalition stability. Adversarial responses are infrequent, and outright rejection is uncommon, given the political significance of the issue. ^20^

Genuinely pro-EU parties in Serbia, such as the Party of Freedom and Justice (SSP), the Green-Left Front (ZLF), and remnants of the Democratic Party (DS), respond to rising Euroscepticism primarily through normative reaffirmation of European values, linking EU integration to democratic consolidation, judicial reform, and media freedom. Rather than engaging in emotional or populist counter-narratives, these actors emphasize institutional and rule-of-law frameworks as the foundation for credible accession. Their criticism is often twofold: directed at the Serbian government for undermining reforms, and at the EU for tolerating democratic backsliding in exchange for regional stability. As such, they adopt what could be described as *adversarial strategies*, positioning themselves in critical opposition to anti-EU narratives while supporting integration - firmly supportive of integration.

The consequences of these responses remain limited due to the fragmentation of the pro-European camp, reduced media access, and weak electoral performance. Unlike mainstream actors in other EU-aspiring states, Serbian pro-European parties cannot effectively shape the national narrative on Europe. Their discourse often resonates only within pro-democracy civil society circles and urban constituencies. One notable example of contested EU-related policy issues. Pro-European actors have attempted to reconcile economic development with democratic and ecological principles, while Eurosceptic actors have framed the issue as evidence of foreign exploitation, elite collusion and threats to national sovereignty. Environmental mobilisation has therefore created opportunities for cross-ideological cooperation, but it has also enabled sovereigntist and anti-EU actors to appropriate ecological grievances.

Mainstream reactions to populist Euroscepticism are constrained by the dominance of the Serbian Progressive Party (SNS), which strategically oscillates between pro-European and sovereigntist discourses. Thus, the response pattern is neither clearly adversarial nor fully accommodative. Instead, SNS neutralizes Eurosceptic actors by co-opting elements of their rhetoric and occupying the full ideological spectrum. It reflects a *variation of the accommodative strategy*, as the party strategically oscillates between pro-European and sovereigntist discourses to maintain political dominance and broaden its electoral appeal. The concrete consequences of Euroscepticism in Serbia are therefore not limited to the direct policy influence of anti-EU parties. They also include the normalisation of selected anti-elite and sovereigntist frames within mainstream political communication.

The coexistence of pro-European and sovereigntist messages may have consequences beyond short-term electoral positioning. By presenting EU integration as strategically desirable while simultaneously portraying the EU as an actor that threatens Serbia’s sovereignty, the governing party can sustain support among voters with divergent preferences. At the same time, this discursive oscillation may blur the perceived meaning and costs of EU membership, contribute to political disengagement and weaken the clarity of the pro-European alternative. The decline in support for EU membership reported in the introduction cannot be attributed to SNS discourse alone. It may also reflect Kosovo-related conflict, distrust in institutions, geopolitical developments and dissatisfaction with the EU’s relationship with the Serbian government. Nevertheless, the pattern is consistent with the possibility that prolonged political ambiguity has contributed to uncertainty and declining enthusiasm about accession. Establishing causal effects would require individual-level longitudinal survey data, which are beyond the scope of this report.

SNS also uses criticism of the EU to deflect accountability, particularly on rule-of-law issues and Kosovo negotiations. Nonetheless, it continues to seek EU funds, participate in accession negotiations and maintain a technocratic administrative structure intended to ensure alignment with EU standards in selected policy areas. This combination of rhetorical resistance and institutional engagement is central to the Serbian form of populist Euroscepticism.

Hard Eurosceptic actors, such as Dveri and, increasingly, the New Democratic Party of Serbia (Novi DSS), have minimal influence on policymaking. Their presence functions more as a symbolic signal to Brussels that public dissatisfaction exists, but they remain excluded from governing coalitions. Overall, Euroscepticism is often a secondary political theme that is mobilised during election campaigns or periods of crisis, but it is ultimately subordinated to broader nationalist narratives, coalition strategies and regime-survival considerations.

The interaction between coalition politics and electoral competition also encourages mainstream actors to adopt selected elements of populist and sovereigntist narratives. The 2023 coalition between Dveri and Zavetnici illustrates cooperation among radical-right actors around nationalism, traditional values, opposition to EU and NATO integration, and closer relations with Russia and China. Zavetnici’s subsequent entry into the SNS-led government in 2024 demonstrates the reverse dynamic. A party that had previously adopted an adversarial and explicitly hard Eurosceptic position moderated its public language after gaining access to governing structures, replacing direct opposition to EU integration with appeals to “national interest” and “strategic neutrality”. This shift reflects an accommodative repositioning aligned with the mainstream coalition narrative. It also demonstrates how participation in government can discipline and instrumentalise populist Euroscepticism, transforming it from a programme of opposition into a more flexible resource within governing-party competition.

## 5. Short conclusion

Populist Euroscepticism in Serbia is state-centric, sovereigntist, and highly instrumental. The Serbian case demonstrates the fluid and performative nature of populist Euroscepticism in non-EU contexts. Unlike EU member states, where Euroscepticism implies contestation from within, in Serbia it often combines Eurosceptic messaging with a pragmatic engagement toward the EU.

Serbian populist actors differ in the intensity and content of their Euroscepticism. Radical-right parties tend to articulate hard Euroscepticism through nationalism, traditionalism and the defence of sovereignty. DJB represented a softer, anti-elite form of Euroscepticism, while SNS combines formal support for EU integration with conditional criticism of EU policies and external pressure. SPS occupies a flexible position shaped by its coalition role. SNS illustrates how a dominant governing party can combine formal support for EU integration with selective sovereigntist criticism in order to appeal to electorally diverse constituencies. The trajectory of Zavetnici further demonstrates how a party’s position inside or outside government can transform its Eurosceptic discourse.

This engagement with the EU does not necessarily reflect a consistent commitment to European values. It also reflects the strategic pursuit of the economic and political benefits associated with EU accession. At the same time, the effects of populist Euroscepticism are not limited to explicit anti-EU parties. Through coalition dynamics, electoral competition and the mainstreaming of sovereigntist frames, selected elements of populist discourse can become part of the broader political agenda. While multiple issues inform Eurosceptic rhetoric, the question of Kosovo represents a central and unifying reference point for all major populist actors, shaping the contours of critique toward both domestic and European actors (Kovačević and Surlić, 2025). It functions not only as a foreign-policy issue but also as a broader symbol of national sovereignty, territorial integrity and resistance to external conditionality. Consequently, references to Kosovo enable populist actors to connect criticism of the EU with opposition to domestic political elites, whom they accuse of making unacceptable concessions or failing to protect Serbia’s national interests.

The lithium controversy represents a more recent and distinct, although related, arena of political contestation. It combines environmental, social, economic and sovereignty-related claims. Recent research shows that anti-lithium mobilisation has connected environmental concerns with public participation, democratic accountability and broader criticism of governance (Markovic, 2025). Research on social-media narratives further demonstrates that lithium activism has drawn on frames ranging from patriotism and local protection to transnational environmentalism, thereby connecting actors across the ideological spectrum (Stepanović, 2024). The issue should therefore be treated not only as an environmental policy dispute but also as a site of contestation over sovereignty, democratic accountability and the role of foreign investors. Its relevance to populist Euroscepticism lies in the way environmental grievances can be incorporated into narratives that pit ordinary citizens against political and economic elites, including domestic decision-makers, foreign companies and EU institutions.

This hybrid model of simultaneous integration and resistance complicates attempts to categorise Serbian political actors neatly. It calls for a nuanced understanding of Euroscepticism in accession countries, where parties may depend on the EU economically and institutionally while using criticism of the EU to construct political legitimacy at home.

## Ethics and consent

Ethical approval and consent were not required.

## Data Availability

No new data were generated or analysed in this manuscript. All data are derived from publicly available sources, which are cited in the reference list.

## References

[1] Andreadis I Vasilopoulou S : The populism-euroscepticism nexus in a contested Europe: The EUPopLink COST Action [version 1; peer review: 3 approved]. *Open Res Europe.* 2026;6:27. 10.12688/openreseurope.22680.1 PMC12910195 41710161

[2] Lorimer M Roch J Kessel S van : Populism, euroscepticism, and the populism–euroscepticism nexus [version 1; peer review: awaiting peer review]. *Open Res Europe.* 2026;6:73. 10.12688/openreseurope.23109.1 PMC13560596 42728954

[3] European Commission: Serbia 2024 Report (SWD(2024) 695 final). 2024. Reference Source

[4] Delegation of the European Union to the Republic of Serbia: EU assistance to Serbia. Reference Source

[5] Delegation of the European Union to the Republic of Serbia: Trade relations between the EU and Serbia. Reference Source

[6] Ministry of European Integration: European orientation of citizens of Serbia: Public opinion poll (December 2022). 2023. Reference Source

[7] Ministry of European Integration: Public opinion research.(accessed March 2026). Reference Source

[8] Regional Cooperation Council: Balkan Barometer 2024: Key findings. 2024. Reference Source

[9] Ministry of Public Administration and Local Self-Government: Register of political parties.(accessed March 2026). Reference Source

[10] Spasojević D : Balancing on a pin: Serbian populists, the European Union and Russia. *The impacts of the Russian invasion of Ukraine on right-wing populism in Europe.* European Center for Populism Studies;2023. p.266–277. Reference Source

[11] Bieber F : Patterns of competitive authoritarianism in the Western Balkans. *East European Politics.* 2018;34(3):337–354. 10.1080/21599165.2018.1490272

[12] Kovačević D Surlić S : Populist patterns in post-conflict settlement: leadership discursive strategies in the Serbia-Kosovo status dispute. *Southeast European and Black Sea Studies.* 2025;1–19. 10.1080/14683857.2025.2565869

[13] Konitzer A : Speaking European: Conditionality, public attitudes and pro-European party rhetoric in the Western Balkans. *Eur. Asia Stud.* 2011;63(10):1853–1888. 10.1080/09668136.2011.618699

[14] Jakšić I Gajić Đ Vlajnić N : How partisan media exposure shaped party evaluations in the 2023 elections in Serbia. *Politički život: časopis za analizu politike.* 2023;26:49–79. 10.18485/fpn_pz.2024.26.4

[15] Dveri: Search results for “Alternative za Nemačku”.(accessed March 2026). Reference Source

[16] FoNet: Podrška za AfD.(accessed March 2026). Reference Source

[17] Plus Online: Predstavnici Srpske stranke Zavetnici prisustvovali međunarodnoj konferenciji u Sofiji “Novi lideri Evrope”i posetili KSB bugarsku građevinsku komoru. 2024. (accessed March 2026). Reference Source

[18] National Assembly of the Republic of Serbia: National Assembly in numbers.(accessed March 2026). Reference Source

[19] City of Belgrade: City Election Commission adopted the decision on preliminary results of the elections for councillors of the Belgrade City Assembly. 2024. (accessed March 2026). Reference Source

[20] Styczyńska N Dajč H : Between the past and the future: Eurosceptic political parties and Serbia. Sondel-Cedarmas J Berti F , editors. *The right-wing critique of Europe: Nationalist, sovereignist and right-wing populist attitudes to the EU.* Routledge;2022. 10.4324/9781003226123

