# Supplementary material for: EUPopLink Country report - Serbia
Source: Open Res Eur. 2026 Sep 16;6:151. Originally published 2026 May 7. [Version 2] doi: 10.12688/openreseurope.23443.2 (PMC13247566; doi:10.12688/openreseurope.23443.2)
Supplement: Peer review history

# Reviewer report: Reviewer response for version 2

**DOI:** 10.21956/openreseurope.27274.r81165

**Published:** 2026-10-10

**Authors:** Valentina Petrović

**Affiliations:** 1 University of Zurich, Zurich, Switzerland

Thank you for the revised version. The comments and suggestions have been addressed in the current version.

Is the case presented with sufficient detail to be useful for teaching or other practitioners?

Yes

Is the work clearly and accurately presented and does it cite the current literature?

Yes

If applicable, is the statistical analysis and its interpretation appropriate?

Not applicable

Are all the source data underlying the results available to ensure full reproducibility?

No source data required

Are the conclusions drawn adequately supported by the results?

Yes

Is the background of the case’s history and progression described in sufficient detail?

Partly

Reviewer Expertise:

NA

I confirm that I have read this submission and believe that I have an appropriate level of expertise to confirm that it is of an acceptable scientific standard.

# Reviewer report: Reviewer response for version 1

**DOI:** 10.21956/openreseurope.25368.r74327

**Published:** 2026-07-08

**Authors:** Valentina Petrović

**Affiliations:** 1 University of Zurich, Zurich, Switzerland

Overall, this is a well-structured and informative country report that provides a useful overview of political parties and Euroscepticism in Serbia. The following comments are intended to strengthen the analytical clarity and conceptual consistency of the report.

**1. Introduction**

 The authors state that: "This analysis concentrates on the post-2012 period, following the Serbian Progressive Party's (SNS) rise to power, a development that significantly reshaped Serbia's political dynamics."

 The rationale for selecting 2012 as the starting point should be explained more explicitly. Although the SNS's rise to power undoubtedly transformed Serbia's political landscape, its initial electoral success was also based on a relatively pro-European platform. Therefore, it is not immediately clear why 2012 represents a decisive break in Serbia's foreign policy or in the relationship between populism and Euroscepticism. The authors should clarify whether the discontinuity concerns foreign policy orientation, domestic political competition, party system transformation, state capture, or another dimension.

**2. Chapter 2**

 The statement that there are "no parliamentary parties that fully embody left-wing populism" requires further clarification. The definition adopted by the authors appears to focus primarily on economic redistribution and anti-capitalism. However, if this definition is retained, it should be explicitly introduced. Alternatively, the authors should acknowledge that some actors (e.g., the Green–Left Front, ZLF) could be discussed in relation to culturally progressive or left-wing politics, even if they do not fit the chosen definition. Similarly, the concept of valence populism should be briefly defined before it is applied to several parties. As this is not a widely familiar category outside the specialist literature, a concise explanation would improve accessibility of the report.

 The authors also state that they focus on the most relevant parliamentary parties. However, the omission of the main parliamentary opposition parties in this second chapter is somewhat surprising, particularly since several of them are discussed later in Chapter 4. A brief explanation of the selection criteria, or a more consistent treatment of opposition parties throughout the report, would improve coherence. Also, The New Democratic Party of Serbia (Novi DSS) is introduced rather abruptly in Chapter 4. Since the party has not previously been discussed, the discussion of the party remains unclear.

**3. Environmental protests**

 The discussion of the environmental protests (particularly those surrounding lithium mining) would benefit from engagement with additional (more recent) academic literature. At least one further reference should be included to support this section and situate it within the broader scholarship on environmental mobilization and political contestation in Serbia.

**5. Populist Euroscepticism & Conceptual discussion**

 The manuscript employs the concept of populist Euroscepticism, but the term is not clearly introduced. While the concept is developed within the EUPopLink framework (Andreadis & Vasilopoulou, 2026), a concise definition early in the paper would help readers unfamiliar with this literature understand how the authors distinguish it from populism and Euroscepticism as separate phenomena.

 Moreover, several terms (e.g *adversarial strategy*, *dismissive strategy*) appear without definition. In addition, some concepts are highlighted in bold while others are not. If this formatting follows the country report template, this should be applied consistently. Otherwise, the use of bold formatting should be reconsidered.

**Minor comments**

- The manuscript would benefit from careful language editing. Some sections read fluently, while others contain stylistic inconsistencies.
- Please check the consistency of references and citation style throughout the manuscript. For example, the citation "(Kovačević and Surlić, 2025)" does not appear to follow the same referencing style used elsewhere.

Is the case presented with sufficient detail to be useful for teaching or other practitioners?

Yes

Is the work clearly and accurately presented and does it cite the current literature?

Yes

If applicable, is the statistical analysis and its interpretation appropriate?

Not applicable

Are all the source data underlying the results available to ensure full reproducibility?

No source data required

Are the conclusions drawn adequately supported by the results?

Yes

Is the background of the case’s history and progression described in sufficient detail?

Partly

Reviewer Expertise:

Political sociology, political behaviour, comparative political economy

I confirm that I have read this submission and believe that I have an appropriate level of expertise to confirm that it is of an acceptable scientific standard, however I have significant reservations, as outlined above.

# Reviewer report: Reviewer response for version 1

**DOI:** 10.21956/openreseurope.25368.r74328

**Published:** 2026-06-08

**Authors:** Jovana Zafirović

**Affiliations:** 1 Institute of Social Sciences, Belgrade, Serbia

This report offers a well-structured and analytically coherent account of populist Euroscepticism in Serbia. The mapping of political actors across the valence/radical right distinction is convincing, and the treatment of how parties shift their EU positions depending on whether they are in opposition or government, which was illustrated effectively by the Zavetnici case, is one of the report's stronger contributions.

 I would suggest two possible minor additions to the authors:

 First, the analysis would benefit from some electoral context. The report discusses parties in terms of their rhetoric and positioning, but rarely situates them by their actual weight in the National Assembly. Knowing which actors command significant voter blocs and which remain marginal would help readers calibrate how consequential each form of Euroscepticism actually is in practice.

 Second, the report argues that SNS strategically co-opts Eurosceptic rhetoric while maintaining a formally pro-EU stance. It would be worth exploring this dynamic a step further by asking what effect this has on voters: does SNS's oscillation between pro-European and sovereigntist messaging contribute to public confusion or disengagement around EU integration? The declining support figures cited at the outset (from 54.7% average to 34% in 2024), already suggest this is a consequential question worth at least briefly reflecting on.

 Overall, I find the report clear and a valuable contribution to the comparative study of populist Euroscepticism in accession countries.

Is the case presented with sufficient detail to be useful for teaching or other practitioners?

Yes

Is the work clearly and accurately presented and does it cite the current literature?

Yes

If applicable, is the statistical analysis and its interpretation appropriate?

Not applicable

Are all the source data underlying the results available to ensure full reproducibility?

No source data required

Are the conclusions drawn adequately supported by the results?

Yes

Is the background of the case’s history and progression described in sufficient detail?

Yes

Reviewer Expertise:

Political Sociology

I confirm that I have read this submission and believe that I have an appropriate level of expertise to confirm that it is of an acceptable scientific standard.

# Reviewer report: Reviewer response for version 1

**DOI:** 10.21956/openreseurope.25368.r74329

**Published:** 2026-05-27

**Authors:** Christian Lamour

**Affiliations:** 1 Luxembourg Institute of Socio-Economic Research (LISER), Esch-sur-Alzette, Luxembourg

I find this report on Euroscepticism and populism in Serbia very clear, particularly in its relevant references to the evolution of parties over time. The references are well integrated into the discussion and a clear categories is mentioned between hard and soft Euroscepticism. The report is both synthetic and sufficiently detailed regarding each of the parties discussed.

 I would suggest two possible additions or revisions to the authors:

 First, the report refers to a series of concepts that are not always clearly defined: populism, sovereignism, authoritarianism, and Euroscepticism. It would be useful for readers to know more precisely how these terms are understood in the report. For example, Euroscepticism among left-wing populist parties may differ significantly from that of right-wing populist parties. Highlighting such nuances and distinctions would add further value to an already strong report.

 Second, Serbian politics is fragmented, as is the case in many EU countries, and I wondered whether alliances between populist and non-populist or between left/right populist parties were considered. Relatedly, it would be interesting to explore how non-populist parties contribute to the mainstreaming of populist parties by reusing their narratives or adopting agendas initially set by populist actors. The report refers to the ambiguity of the political mainstream; perhaps it would strengthen the analysis to provide a few examples or policy issues on which mainstream parties adopted populist positions concerning the EU integration.

 These are relatively minor suggestions. Overall, I found the report very interesting.

Is the case presented with sufficient detail to be useful for teaching or other practitioners?

Yes

Is the work clearly and accurately presented and does it cite the current literature?

Yes

If applicable, is the statistical analysis and its interpretation appropriate?

Not applicable

Are all the source data underlying the results available to ensure full reproducibility?

Yes

Are the conclusions drawn adequately supported by the results?

Yes

Is the background of the case’s history and progression described in sufficient detail?

Yes

Reviewer Expertise:

NA

I confirm that I have read this submission and believe that I have an appropriate level of expertise to confirm that it is of an acceptable scientific standard.
